# Abdominal Pain of Functional Gastrointestinal Disorders in Dietary Diversity Patterns and Its Determinants among Healthy Adults in Jimma City, Southwest Ethiopia

**DOI:** 10.4314/ejhs.v33i6.13

**Published:** 2023-11

**Authors:** Belay Zawdie, Kalkidan Hassen Abate, Dessalegn Tamiru, Tefera Belachew

**Affiliations:** 1 Department of Biomedical Sciences, Jimma University, Jimma, Ethiopia; 2 Department of Nutrition and Dietetics, Jimma University, Jimma, Ethiopia

**Keywords:** FGIDs, Dyspepsia, IBS, Dietary diversity, Lipid profile

## Abstract

**Background:**

Functional Gastrointestinal Disorders (FGIDs) and their risk factors vary from region to region. Therefore, this study aimed to determine the prevalence of abdominal pain of FGIDs in different dietary diversity score (DDS) and its determinant factors among adults in Jimma City, Southwest Ethiopia.

**Methods:**

A community-based cross-sectional study was conducted from July 17 to October 27, 2019. The study included systematically selected healthy adults aged ≥ 18years. Data were collected on gastrointestinal symptoms (Rome III), and DDS (24-dietary recall).

**Results:**

Of 865 healthy adults, the prevalence of abdominal pain symptoms co-occurrence was 168(19.4%), dyspepsia, 152(17.6%) and IBS, 133(15.4). Similarly, the co-occurrence was distributed as 81(9.4%) in middle, 64(7.4%) in high and 23(2.6%) in low DDS groups. Although this distribution was different in the DDS groups, it is not significantly associated. With potential confounders adjusted, the behavioral factors associated with the co-occurrence with an AOR (95% CI) were khat chewing: 7.37 (1.76 - 30.87), drinking alcohol: 3.24 (1.15 - 9.18), sedentary life: 12.28 (3.19 - 48.40) and less physical activity: 4.44 (1.43-13.75). Moreover, elevated TAG: 5.44 (2.78 - 8.10), elevated LDL: 4.26 (1.61-11.29), central obesity: 2.78 (1.08 -7), low HDL 5.89 (2.22-15.60), positive H.pylori stool test: 2.7 (1.86 -7.72), being diabetic: 2.7 (1.79 -7.79) and hypertensive: 2.79 (1.08 - 7.14) were associated with the co-occurrence.

**Conclusion:**

Abdominal pain and FGIDs had significant distribution among adults in Jimma City. Therefore, early screening and managing FGIDs in the community is recommendable.

## Introduction

Functional gastrointestinal disorders (FGIDs) are a heterogeneous group of disorders caused by abnormal functioning of the gastrointestinal tract (GIT). They cause chronic symptoms throughout the gut (irritable bowel syndrome (IBS), functional constipation (FC), diarrheal, functional dyspepsia, cyclic vomiting syndrome (CVS), nausea, bloating, and functional diarrhea), all of which are made worse by maladaptive patient behaviours, stress and psychological comorbidity (1,2). On top of that, FGIDs are becoming a major public health concern because they are becoming common asymptomatically, impeding daily activities, and causing a significant social and economic burden in the community (3).

Although FGIDs had potentially negative effects on health, early screening and identifying the potential risk and triggering factors have received relatively little attention in developing countries (3,4). This is because FGIDs refer to recurrent gastrointestinal symptoms without any evident organic or structural abnormalities revealed by using either x-rays, CT-scan, blood tests, or endoscopic exams (5). Therefore, those many routine medical tests trying to diagnose FGIDs mostly display essentially non-disease results (1). Due to those limitations with the medical tests, the Rome III criteria or algorithm for the diagnosis of FGIDs has been developed by the Rome Foundation Board, and it has been repeatedly tested and carefully validated to employ for the early screening of FGIDs (6).

Despite many studies that have improved the theoretical understanding of multiple FGIDs, clinical management and identifying triggering factors have still been recognized as major challenges in developing countries. Food plays an important role in the pathogenesis of FGIDs, and dietary interventions offer an opportunity for the patients to manage their own symptoms without relying on pharmaceutical therapy (7, 8).

Although dietary role in pathogenesis and triggering of FGIDs is difficult and expensive to conduct, it is feasible to look at the distribution of FGIDs in different ditiary diversity patterns and trigerring factors (9). One major reason for difficulty could be attributed to physicians' lack of understanding of FGIDs patients' perceptions and their demands. Furthermore, illness perceptions can vary greatly from one to another, even in those with same symptoms (10).

Generally, the findings of numerous studies about FGIDs and related issues are restricted to children, adolescents, and developed countries (11,12). However, there are scarce evidences about awareness of adults with multiple FGIDs and seeking medical help in developing countries like Ethiopia. Therefore, this study aimed to detect abdominal pain of FGIDs and determinant triggering factors among apparently healthy adults in Jimma City, Southwest Ethiopia.

## Materials and Methods

**Study area, design and period**: A community-based cross-sectional study was carried out in six kebeles of Jimma city, Southwest Ethiopia, from July 17 to October 27, 2019. Jimma city is one of the oldest and historic cities in Ethiopia. It is in Oromia National Regional State, in Jimma zone, located 352 kms to Southwest of the Ethiopian capital, Addis Ababa. Geographically, the city is located at 7º40′24.47″N latitude and 36º 5′4.95″ E longitude. Jimma city is the most important city in Southwestern Ethiopia and the study area has been chosen because of the fast rate of urbanization and paradigm shift to use the processing food because of it is a cash crop area. The city has a total population of 195,443, of whom 97,629 are men and 97,814 women with an area of 100.2 km2 (Central Statistical Agency of Ethiopia 2007). The number of households in the 17 kebeles of Jimma city in 2018 was 45,126 (13).

**Sample size and sampling techniques**: The sample size was determined using the single population proportion formula, considering 48.4% of prevalence of dyspepsia (14), confidence interval (CI), margin of error/precision, and design effect to be 95%, 3% and 1.5, respectively:


$$n=(\frac{z\alpha }{2}{)}^{2}\;p\frac{\left(1-p\right)}{d^{2}}=\frac{\left(1.96{)}^{2}(0.484x0.516\right)}{(0.03{)}^{2}}=1066$$

The final sample size of adults involved in this study was nearly equal to 865 with response rate of 81.14%. First, 1066 total sample size was allocated proportionally to the size of six selected kebeles (Ginjo Guduru, Awetu Mendera, Mendera Kochi, Ginjo, Bocho Bore, Hermata Markato) based on the total number of households in each kebele. The total number of households from those six kebeles was 25,122 (13). The first household was selected by simple random sampling and more than one study participants selected from the same house by lottery method. Then, the next household was selected through systematic sampling technique that is every K^th^ interval household (which was calculated for each kebele). In a case when the study participants were not able to be interviewed for some reasons (e.g. absenteeism), three visit-rule was used to interview the respondent, and if were not accessible after all, they were considered as non-respondents.

**Study population and selection criteria**: This study targeted apparently healthy adults aged ≥ 18 years old and resides in Jimma city for at least six months before the data collection. However, adults who had physical deformity (kyphosis, scoliosis), early confirmed with any various upper and lower abdominal pains of the FGIDs and common NCDs (diabetes mellitus, CVD, mental illness, hypertensive, metabolic syndrome, dyslipidemia) and pregnant women were excluded. Moreover, participants who had been taking treatment against H. *pylori* infection within three months before the current study and those who used proton pump inhibitors or H_2_-blockers for more than two weeks before this study's enrollment were also not selected.

**Data collection procedure and measurements**: All study participants were interviewed using a well-structured questionnaire adapted from WHO STEPS questionnaire (14) and customized to the local context based of the study. The questionnaire was first written in English, translated into Amharic and Afaan-Oromoo languages by language experts, and then re-translated back into English by a panel of professionals to check for consistency.

**Variables Measurement Procedures and Their Operational Definitions: -**

**Socio-demographic and behavioral characteristics**: The data for socio-demographic and behavioral characteristics were collected through face-to-face interviews. According to WHO criteria, an adult person should do at least 150 minutes of moderate-intensity work or 75 minutes of vigorous-intensity work per week. Based on this, if participants self-reported physical activity did not fit with the WHO recommendation, participants were categorized as physically inactive (15). Similarly, sedentary behavior was measured by asking about the time spent sitting during a typical week. Based on that, the responses were classified as <4 hours/day (not under sedentary) and ≥4 hours/day (sedentary) (16). Moreover, khat chewing, tobacco smoking and alcohol consuming were defined as self-reported person who is currently chewing khat, smoking tobacco products and consuming more than one alcoholic drink per week, respectively (17).

**Anthropometric measurements**: Anthropometric measures, including weight, height, waist-circumference (WC), and hip-circumference were recorded using standardized procedures. Then, body mass index (BMI) was calculated as weight in kilogram divided by height in meter squared. BMI< 18.5 kg/m^2^ is considered underweight, 18.5–24.9 kg/m^2^ is normal, 25–29.9 kg/m^2^ is overweight, and ≥30 kg/m^2^ is obese (18). WC values > 94cm for men and >80cm for women were considered high according to the WHO's recommendation (19).

**Blood pressure measurements**: Blood pressure (BP) was measured in sitting position from the right arm and in triplicate using an Aneroid Sphygmomanometer with small, medium, and large cuff sizes (20) as fit to the subjects after five minutes of rest. The subsequent measurements were done five minutes apart. According to the WHO recommendation, pre-hypertension is defined as a systolic BP of 120–139 mmHg and a diastolic BP of 80–89mmHg. On the other hand, hypertension is defined as systolic BP of ≥140mmHg and/or diastolic BP of ≥90 mmHg (21).

**Screening upper and lower FGIDs**: Rome III diagnostic criterion for screening adults with the various FGIDs were adopted and pre-testing was performed to validate the criteria. The Rome III diagnostic screening for upper abdominal pain (functional dyspepsia) use bothersome, postprandial fullness, early satiation, epigastric pain and burning symptoms. Adults were diagnosed as functional dyspepsia (FD) if all the above criteria are fulfilled for the last 3 months with symptom onset at least 6 months prior to this diagnosis. Similarly, the Rome III diagnostic screening for adults with lower abdominal pain/irritable bowel syndrome (IBS) was using relief with defecation, onset associated with a change in frequency and appearance of stool symptoms. Adults were diagnosed as IBS if they had repeated abdominal pain or discomfort for at least 3 months in the previous 6 months, with 2 or more of the above symptoms (22). Moreover, for the purpose of this study, adults who fulfilled with both upper and lower abdominal pain symptoms considered as co-occurrence of the upper and lower abdominal pain symptoms of the FGIDs.

**Dietary survey and dietary diversity score (DDS)**: Six data collectors and six health extension workers were recruited and trained for 2 days, a week before the final data collection date. Then, data collectors were paired with local health extension workers from each six selected kebeles and allowed to collect detailed information on the varieties of food consumption using three consecutive 24-hour dietary recalls (including two weekdays and one weekend day).

According to the dietary individual or women dietary diversity guidelines (23), all food items (16) were categorized into 9 groups which were meat, grains, vegetables, fruits, beans, eggs, fish, dairy and oil. If a participant consumed any food item from any of the above nine mentioned categories, he/she would get one point in that food category. However, if not, he/she would be scored zero. As assumption, consuming different foods from the same category would not count repeatedly. The total score would be the sum scores of the nine food groups and the maximum score could go up to 9. Therefore, for the purpose of this study the DDS was categorized into low DDS (≤ 3 food groups), medium DDS (4 and 5 food groups), and high DDS ( ≥6 food groups) (23).

**Stool sample collection and detection of Helicobacter pylori (H. *pylori*)**: About nearly 20g of fresh stool was collected from each participant in sterile and screw-capped containers. Fresh stool specimen was tested using one step stool antigen test (Zhejiang Orient Gene Biotech CO., LTD, China) with 94.9%–100% sensitivity and 95–100% specificity (24). Test results were read and interpreted according to the manufacturer's instructions and protocol by laboratory experts. The test kit is validated and approved by the ministry of health and quality controlling agency of the country and is currently used as the gold standard for the diagnosis of *H. pylori* infection in the country.

**Determination of blood glucose level and lipid profiles**: Five-milliliter of venous blood was collected from each study participant to determine either random or fasting blood glucose levels or lipid profiles. Serum was carried out in ABX Pentra 400 Automated Chemistry Machine (Horiba ABX SAS, 34184 Montpellier, France) at JMC clinical chemistry core laboratory to determine those serum biomarkers. However, the low density lipoprotein cholesterol (LDL-cholesterols) level was calculated by using the Fried Wald formula (25).

The diagnosis of diabetes mellitus (DM) was based on the International Diabetes Federation (IDF) classification criteria with fasting blood glucose (FBG) of ≥ 126 mg/dl or random blood glucose (RBG) of ≥200 mg/dl considered as positive for DM; Pre-diabetes as if FBG: 100 mg/dl ≤ FBG ≥ 126 mg/dl or RBG: 140 mg/dl ≤ RBG > 199 mg/dl (26). Moreover, total cholesterol (TC) ≥ 200 mg/dl was considered as hypercholesterolemia and < 200 mg/dl as normal. The optimal level of LDL-c was considered as < 100 mg/dl, but otherwise, defined as elevated LDL-c. Triacylglycerol (TAG) level was considered as normal when it was < 150 mg/dl or hypertriglyceridemia when it was ≥ 150 mg/dl. Similarly, HDL-c was considered desirable if it was > 40 mg/dl for men and > 50 mg/dl for women; otherwise, it was classified as lower HDL-c (27).

**Quality assurance, data processing and statistical analysis**: First, the data were checked for completeness and consistency and double-entered EPI data software version 3.1. Then, the data were exported to SPSS for windows version 27 for analysis. After normality and multi-collinearity were checked, descriptive data were presented as frequency and percentage while continuous data were presented as mean ± SD, and frequency (%). Tables and figure were generated for categorical variables. Bivariate and multivariate logistic regression analyses were used to assess the association between independent variables and the outcome. All the data were analyzed at 95% CI, and with a value of p<0.05 was considered to be statistically significant for all analyses.

**Ethical approval and consent to participate**: The study protocol was reviewed and approved by the Institutional Review Board of Institute of Health, Jimma University (Ref. No: IHRPGD 595/2019). A letter of support was obtained from Jimma city and communicated to the selected kebele administrations. Besides, before data collection, written informed consent was obtained from each study participant. Additionally, for participants who could not read the informed consent form, the document was orally presented to them in the presence of an independent witness. After informed consent was obtained from them and it was approved by the ethics committee of Jimma University, the Institute of Health Review Board. All the participants' information was kept confidential using a coding system. Participants identified with cases were advised and linked to nearby health facilities for further investigation and management. All methods were carried out following relevant guidelines and regulations.

## Results

**Socio-demographic and behavioral characteristics**: A total of 865 apparently healthy adults (≥ 18 years old) were enrolled. Of the sampled study participants, 450(52.0%) were males. Of the study participants, for smoking cigarettes, drinking alcohol and chewing khat, the distributions were 71(8.2%), 150(17.3%) and 315(36.4%), respectively. The majority (91.1%) of the study participants were using iodized salt. Of the study participants, 70.4% had frequent practice of drinking hot tea and coffee. Nearly 660(71.3%) of the participants were using vegetable oil. Moreover, participants with less physical activity and sedentary life accounted for 308(45.6 and 521(60.3%), respectively (Table 1).

**Table 1:** Frequency distribution of socio-demographic and behavioral characteristic and practices among healthy adults in Jimma City, Southwest Ethiopia, 2019(n=865)

Variable and Category	Frequency (%)
**Sex**	
Male	450 (52.0)
Female	415 (48.0)
**Age group(yrs.)**	
<35	378(43.7)
≥35	487(56.3)
**Educational status**	
Cannot write and read	172(19.9)
Primary School	422(48.7)
Secondary School	213(24.6)
Diploma and above	59(6.8)
**Marital status**	
Single	125(14.4)
Married	618(71.3)
Divorced	75(8.7)
Widowed	35(4.0)
Separated	12(1.4)
**Occupations**	
Unemployed	121(14.0)
Student	52(6.0)
Housewife	157(18.1)
Daily laborer	128(14.8)
Merchant	88(10.1)
Gov. Employee	181(20.9)
Priv. employee	138(15.9)
**Family size**	
1-2	140(16.1)
3-4	444(51.4)
>5	281(32.5)
**Annual Income (ETB)**	
≤10,000	366(42.2)
10,001–49,999	410(47.3)
≥50,000	89(10.3)
**Smoking cigarette**	
Yes	71(8.2)
No	794(91.8)
**Alcohol conception**	
Yes	150(17.3)
No	715(82.7)
**khat chewing**	
Yes	315(36.4)
No	550(63.6)
**In hot drink tea trend**	
Yes	646(74.5)
No	219(25.3)
**In hot drink coffee trend**	
Yes	573(66.3)
No	292(33.7)
**Type of oil or fat used**	
Vegetable oil	660(71.3)
Butter or ghee, margarine	191(22.1)
Not in particular	14(1.6)
**Iodized salt used practice**	
Always	797(91.1)
Often	30(3.5)
Sometimes	38(4.4)
**Level of physical activity**	
Sufficient	308(35.6)
Insufficient	557(64.4)
**Sedentary behavior**	
<4 hours/day	344(39.7)
≥4 hours/day	521(60.3)

**Physical and clinical characteristics:** Of the study participants, 238(27.4%) were overweight and obese whereas 215(24.8%) had central obesity. On the other hand, the study participants diagnosed with hypertension and diabetes mellitus were 192(22.2%) and 171(19.8%), respectively. Moreover, lipid profile derangements were: 95(11.1%) elevated TC, 407(47.0%) decreased HDL-c, 265(30.6%) elevated LDL-c and 241(27.9%) elevated TAG (Table 2). The prevalence of upper and lower abdominal pain co-occurrence of the FGIDs was 168(19.4%) whereas the prevalence of upper abdominal pain (dyspepsia) alone was 152(17.6%). Similarly, the prevalence of lower abdominal pain (IBS) alone was, 133(15.4) (Table 2).

**Table 2:** Physical and biochemical measurements characteristic among healthy adults in Jimma City, Southwest Ethiopia, 2019 (n=865)

Variable and Category	Frequency (%)
**Body mass index**	
Underweight	127(14.6)
Normal	500(57.9)
Overweight	164(19.0)
Obese	74(8.5)
**Waist circumferences**	
Non-Central Obese	650(75.2)
Central obese	215(24.8)
**Hypertension**	
Yes	192(22.1)
No	673(77.8)
**Pre-and diabetes milieus**	
Yes	171(19.8)
No	694(80.2)
**Lipid profiles**	
TC	
<200 mg/d	770(87.9)
≥200 mg/d	95(11.1)
HDL-c	
Low	407(47.0)
Normal	458(53.0)
LDL-c	
<100	600(69.3)
mg/dl	
≥100	265(30.6)
mg/dl	
TAG	
<150 mg/dl	624(72.1)
≥150 mg/dl	241(27.9)
**Multiple FGIDS**	
Yes	
FGIDs (co-occurrence)	168(19.4)
FD alone	152(17.6)
IRB alone	133(15.4)
No	412(47.6)

H. pylori= Helicobacter pylori; Functional Gastrointestinal Disorders; FD=Functional Dyspepsia; IBS= Irritable Bowel Syndrome; TC: Total Cholesterol; HDL-c: High Density Lipoprotein-Cholesterol; LDL-c: Low Density Lipoprotein-Cholesterol; TAG: Triacylglycerol.

**Figure 1:**
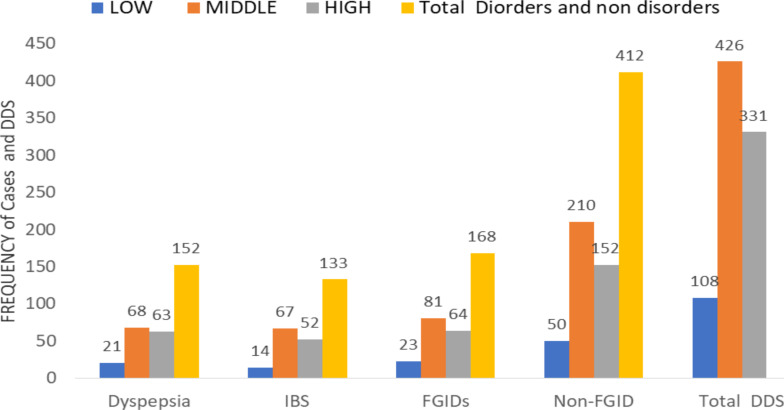
Magnitude of multiple FGIDs among different Dietary Diversity Score (DDS) group among health adults in Jimma City, Southwest Ethiopia, 2019(n=865) FGIDs= Functional Gastrointestinal Disorders; FD=Functional Dyspepsia; IBS= Irritable Bowel Syndrome; DDS=Dietary Diversity Score; LDDS=Low Dietary Diversity Score; MDDS=Middle Diversity Score; HDDS=High Dietary Diversity Score

**FGIDs and dietary diversity scores (DDS)**: The prevalence of upper and lower abdominal pain co-occurrence of the FGIDs was distributed as 81(9.4%) in middle, 64(7.4%) in high and 23(2.6%) in low DDS groups. Similarly, the prevalence of dyspepsia and IBS alone each was distributed from the middle to low DDS groups as similar pattern of upper and lower abdominal pain symptoms co-occurrence of the FGIDs (Figure 1).

**Factors associated with co-occurrence of upper and lower abdominal pain symptoms**: As shown in Table 3, with potential confounders adjusted, the factors associated with pain symptoms co-occurrence of the FGIDs with an AOR (95% CI) were khat chewing: 7.37(1.76 - 30.87), currently drinking alcohol: 3.24(1.15 - 9.18), low-income wealth index: 2.63(1.16 - 5.96), using butter or ghee and margarine: 6.79(2.82-16.37). Moreover, Sedentary life: 12.28 (3.19 - 48.40), being less physical activity: 4.44 (1.43-13.75), elevated TAG: 5.44 (2.78 - 8.10), elevated LDL-C: 4.26(1.61-11.29), central obesity: 2.78 (1.08 -7.14), decreased HDL-c: 5.89 (2.22-15.60), positive *H. pylori* stool antigen test: 2.7 (1.86 -7.72), being diabetic: 2.7 (1.79 -7.79) , and hypertensive: 2.79 (1.08 - 7.14) were significantly associated with the co-occurrence of the FGIDs (Table 3).

**Table 3:** Bivariate and multivariate regression analysis to identity risk factors associated with upper and lower abdominal pains co-occurrence of the FGIDs among healthy adults in Jimma City, Southwest Ethiopia 2019(n=865)

Variables	Co-occurrence of FGIDs	COR,95%CI	AOR,95%CI	*P- value*
**Khat Chewing**				
Yes	128	4.43(2.13, 9.24)	7.37(1.76,30.87)	***0.006***
No	40	1	1	
**Currently alcohol drinking**				
Yes	89	6.56(3.68, 11.69)	3.24(1.15,9.18)	***0.027***
No	79	1		
**Trend of drinking coffee, tea and others in hot**				***0.005***
Yes	130	4.53(2.23, 10.24)	5.37(1.76,28.87)	
No	38	1	1	
**Wealth index**				
middle income	66	1		
low income	82	2.05(1.27-3.29)	2.63(1.16-5.96)	***0.005***
higher income	20	1.37(0.66-2.85)	1.09(0.32-3.68)	
**Type of oil or fat often used**				
Vegetable oil	76	1	1	
Butter or ghee, margarine	89	6.63(3.67-11.96)	6.79(2.82-16.37)	
None in particular	3	4.47(0.83-24.10)	1.87(0.17-20.46)	***0.004***
**Sedentary life**				
≥ 4hours/day	113	6.24(3.12- 12.48)	12.28(3.19-48.40)	***0.001***
<4hours/day	55	1	1	
**Physical activity**				
Yes	61	1	1	
No	107	8.01(3.96-16.54)	4.44(1.43-13.75)	***0.027***
**TAG (mg/dl)**				
Elevated ≥ 150	119	4.44(2.78-7.10)	5.44(2.78-8.10)	
Normal <150	49	1		***0.001***
**Waist circumference(cm)**				
Central obese	128	3.65(2.27-5.88)	2.78(1.08-7.14)	***0.034***
Non obese	40	1	1	
**LDL-c (mg/dl)**				
≥ 100 High	105	8.47(4.98-14.39)	4.26(1.61-11.29)	
<100 Normal	63	1	1	***0. 004***
**HDL-c (mg/dl)**				
Low <40	111	9.07(5.34-15.23)	5.89(2.22- 15.60)	
Desirable ≥40	57	1	1	***0.001***
**Hypertension**				
No	36	1	1	***0.032***
Yes	132	3.68(2.17-5.89)	2.79(1.08-7.14)	
**Diabetes mellitus**				
No	48	1	1	***0. 033***
Yes	120	3.72 (2.67-8.67)	2.7(1.79-7.79)	
**H. *pylori* stool antigen test**				
Negative	46	1	1	
Positive	122	3.8 (2.86-8.72)	2.7(1.86-7.72)	***0. 035***

FGIDs= Functional Gastrointestinal Disorders; FD=Functional Dyspepsia; IBS= Irritable Bowel Syndrome; TC: Total Cholesterol; HDL-c: High Density Lipoprotein-Cholesterol; LDL-c: Low Density Lipoprotein-Cholesterol; TAG: Triacylglycerol.

## Discussion

This is the first population-based study among adults aged ≥ 18 years old in urban community of southwest Ethiopia. In Ethiopia, the spectra of dyspepsia FGIDs was investigated in 2018, but the study was limited to hospital outpatients (13). Furthermore, most of the previous studies had been limited to children and adolescents and developed countries (11, 12).

In our study, the magnitude of the co-occurrence of upper and lower abdominal pain symptoms of the FGIDs was 19.4%. This result lies in between the overall results of FGID prevalence rates ranging from 9.9% to 29% (28) and in line with the study on the men in the Korean army, 18% (29). However, this prevalence is less than the finding among college students in northern India, 26.9% (30), and male civil pilots in China, 37.4% (9). On the contrary, it was higher as compared with the findings from residents of Olmsted County, MN, 10% (31). Moreover, the distribution of functional dyspepsia (FD) alone was 17.6% in the current study. This is in line with the findings from the global perspective of uninvestigated dyspepsia, which varied between 7% - 45% (32), but less than the result of the study done in northeast Ethiopia, 48.4% (14). This study is also relatively similar to the study done on the healthy US adult population, 21.2% (33), but higher than the findings of a study done in North India, 15.2% (30). Likewise, the magnitude of irritable bowel syndrome (IBS) alone in the current study was 15.4%. This finding lies between the results of the systematic review done in Serbia, 9 to 23% (34). Nevertheless, it is contrary to the results of the study done in North India (30), and the US (33). The inconsistency of the results in magnitude might be attributed to variations in behavioral factors, dietary-related factors, lifestyle factors, sociocultural, and psychological issues (30). Moreover, the rate of gastrointestinal infection relating to H. *pylori*, and the stability of the gut micro-biota (14).

The present study demonstrated that the prevalence of upper and lower abdominal pain co-occurrence of the FGIDs was found to have a higher distribution in the middle, followed by the higher and less in the low DDS groups. Similarly, the prevalence of dyspepsia and IBS was found to have a higher percentage distribution in the middle DDS group as compared with the low DDS group, as shown in Figure 1 above. Although, the varied distributions of various groups of FGIDs vary in different DDS groups, there was no significant association found between DDS groups and various groups of FGIDs. This is in line with the findings among Iranian adults (35). On the other hand, many studies in the world have shown that higher DDS contributes to higher energy for obesity (36), which is consequently associated with the progression of various groups of FGIDs (37).

The current study revealed a higher upper and lower abdominal pain co-occurrence of the FGIDs observed in ages ≥ 35 years old (56.3%) as compared with age less than 35 (43.6%), but the distribution was not statistically significant. This finding was contrary to the study done in China (9), but the data were collected via the Internet in 24 countries, personal interviews in 7 countries, and it was concluded that FGIDs decrease with age (38). Similarly, the current finding showed that the prevalence of upper and lower abdominal pain co-occurrences of the FGIDs was higher in males (52.2%) than in females (48.0%), but it was statistically insignificant. This finding is similar with the study done in Olmsted Country, MN (31). Moreover, the study done in Northern India (30) and the other 33 countries from 6 continents revealed the highest prevalence of FGIDs in females than in males (38). In the current study, the prevalence of FGIDs in adults with BMI > 25kg/m^2^ was higher as compared to BMI ≤ 25kg/m^2^, and this is in line with the studies done in North India (30), but both findings revealed as statistically insignificant association. On the contrary, the study done in China (9) showed that the prevalence of FGIDs in BMI ≤ 25kg/m^2^ was higher than participants with a BMI > 25kg/m^2^ and the association was found to be statistically insignificant.

In the current study, higher prevalence of FGIDs among iodized salt users as compared with non-or less iodized salt users. However, it was not significantly associated with the FGIDs in the recent study but in China (9) findings revealed that high-salt food patterns are twice as likely to influence FGIDs as compared with low-salt food patterns. Similarly, chewing khat practice, drinking trend of hot tea and/or coffee, and alcohol conception practice are significantly associated with upper and lower abdominal pain symptoms co-occurrences of the FGIDs, as shown in Table 3. This is agreed with caffeine, and alcohol worsens reflux symptoms and the risk of functional dyspepsia (39). However, some findings disagree with the associations (13). Moreover, in the current study, significantly associated distributions of upper and lower abdominal pains co-occurred with the FGIDs with fewer physical activities and sedentary as per the recommendation of WHO (15). Poor physical activity was an independent predictor of the FGIDs (30).

In the current study, increased central obesity, dyslipidaemia, being diabetic, hypertensive, and positive H. pylori stool antigen test were significantly associated (all p < 0.05) factors with upper and lower abdominal pains co-occurrence of the FGIDs in the study community as presented in Table 3. The statistically significant association of diabetes mellitus with upper and lower abdominal pain co-occurrence of the FGIDs is consistent with the findings of studies done in Australia (40). Moreover, the significant association of hypertension and dyslipidaemia with abdominal pain co-occurrence of the FGIDs revealed in this study transitivity links with obesity and non-communicable diseases (NCDs) (40).

In our study, upper and lower abdominal pains symptom co-occurrence of the FGIDs was nearly three times more likely to occur among participants confirmed with H. *pylori* positive as compared with negative. Particularly, the occurrence of dyspepsia was highly associated with H. *pylori* infection (13).

In general, the current community-based study is useful to understand various abdominal pain symptoms of the FGIDs asymptomatically existing in the community, and there are various factors in the study community associated with the pain symptoms. Moreover, this finding is useful as a benchmark for researchers and health professionals to look at the cause abdominal pain and reverse its impacts.

Despite much strength this study had, it also had some limitations. Since the study was a single-centred cross-sectional finding, it is not used to infer national realities. Moreover, it did not also include all age groups. It had also the limitation of telling the cause-and-effect relationship of the variables with health-related outcomes and economics.

In conclusion, unrecognized FGIDs were relatively high among the study community. Being female, having a sedentary lifestyle, having physical inactivity, having a higher BMI, higher central obesity, elevated TC, a higher TAG, higher LDL, lower HDL, having diabetes mellitus, being hypertensive, and having positive H. pylori stool antigen were found to be predictors of FGIDs qualified for both upper and lower abdominal pain symptoms. There is no statistically significant correlation between FGIDs and DDS. Thus, awareness needs to be created among the community to practice regular physical activity and maintain a normal body weight. Additionally, screening of FGIDs should be promoted for early detection, prevention and treatment.
